# Association between distance to nearest supermarket and provision of fruits and vegetables in English nurseries

**DOI:** 10.1016/j.healthplace.2017.05.018

**Published:** 2017-07

**Authors:** Thomas Burgoine, John A. Gallis, Tarra L. Penney, Pablo Monsivais, Sara E. Benjamin Neelon

**Affiliations:** a UKCRC Centre for Diet and Activity Research (CEDAR), MRC Epidemiology Unit, University of Cambridge School of Clinical Medicine, Box 285, Institute of Metabolic Science, Cambridge Biomedical Campus, Cambridge CB2 0QQ, UK; b Department of Biostatistics and Bioinformatics, Duke University Medical Center, 2424 Erwin Road, Suite 1102 Hock Plaza, Durham, NC 27710, USA; c Duke Global Health Institute, Duke University, 310 Trent Hall, Durham, NC 27710, USA; d Department of Health, Behavior and Society, Johns Hopkins University, 624 N Broadway, Baltimore, MD 21205, USA

**Keywords:** Childcare, Nurseries, Preschool, Supermarket proximity, Fruit and vegetable provision, Geographic information systems, Nutrition in Nurseries study, England

## Abstract

With 796,500 places available for children in England, pre-school nurseries could serve as an important setting for population-wide dietary intervention. It is critical to understand the determinants of healthy food provision in this setting, which may include access to food stores. This study examined the association between objective, GIS-derived supermarket proximity and fruit and vegetable serving frequency, using data from 623 English nurseries. Overall, 116 (18%) nurseries served fruits and vegetables infrequently (<2–3 times/week), but provision differed by supermarket proximity. In adjusted multivariable regression models, nurseries farthest from their nearest supermarket (Q5, 1.7–19.8 km) had 2.38 (95% CI 1.01–5.63) greater odds of infrequent provision. Our results suggest that supermarket access may be important for nurseries in meeting fruit and vegetable provision guidelines. We advance a growing body of international literature, for the first time linking the food practices of institutions to their neighbourhood food retail context.

## Highlights

• We examined the relationship between nursery supermarket access and the provision of fruits and vegetables to children.
• We used data from a representative sample of 623 English nurseries, alongside objectively measured supermarket proximity.
• 18% of nurseries served fruits and vegetables infrequently, but this differed systematically by supermarket proximity.
• Nurseries farthest from their nearest supermarket were significantly less likely to serve fruits and vegetables frequently.
• Supermarket access may be important for nurseries in meeting guidelines for the provision of fruits and vegetables.

## 1 Introduction

By the time UK children enter primary school at age four, over one fifth are already overweight or obese (Lifestyle Statistics Team, 2015). These excess levels of adiposity tend to track into adolescence and adulthood (Singh et al., 2008, Dehghan et al., 2005). The risk of weight gain and obesity can be reduced through healthy eating. In particular, regular fruit and vegetable consumption, especially for those under the age of five, supports healthy growth and development (Gardner et al., 2009, Feinstein et al., 2008, Wiles et al., 2009), and encourages a taste for healthy food in the long term (Carruth et al., 1998). Yet the majority of children aged 2–10 years do not consume the recommended five portions of fruits and vegetables per day (Nelson et al., 2007, Glynn et al., 2005). Thus, there is a need to better understand the determinants of fruit and vegetable consumption *prior* to children entering primary school.

Pre-school nurseries represent an important setting for population-wide dietary intervention (Osei-Assibey et al., 2016). The number of registered places available in early years childcare in England has been steadily increasing, with 796,500 places available in nurseries in 2013 (Brind et al., 2014). The provision of foods in schools and nurseries is strongly linked to their consumption (van der Horst et al., 2007, French and Stables, 2003, Ball et al., 2008). Healthy nursery practices serve as an opportunity to support parents in encouraging healthy eating at home (Children's Food Trust, 2010a). Further, healthy eating in an educational context such as a nursery can also be especially effective in reducing incidence of ‘fussy’ eating, as well as issues related to the introduction of novel, healthy foods (Carruth et al., 1998). As children attend nurseries from across the socioeconomic spectrum, establishing healthy eating practices may also help to reduce health inequalities across the lifecourse (Marmot, 2010).

It is important to understand the barriers and enablers to provision of healthy food in this setting. The majority of meals provided in nurseries are prepared in-house, by dedicated cooks and kitchen staff, who often source their own ingredients (Children's Food Trust, 2010a). Therefore, access to food stores may be an important contributor to the healthfulness of the foods served. Nurseries report using a range of different types of food outlets for their grocery shopping (Children's Food Trust, 2010b), including wholesalers, convenience stores, specialty food outlets such as butchers and fishmongers, as well as supermarkets (Children's Food Trust, 2010a), which offer a variety of fresh, healthy produce at a range of price points. Nurseries often cite the cost of healthy food as a major barrier to provision (Children's Food Trust, 2010b), with food and drink spending per child per day in English nurseries reported to be as a little as £1 (Parker et al., 2011). As a result, access to a supermarket, as a marker of access to a range of affordable, healthy produce, may relate to the provision of healthy foods within nurseries. To our knowledge, no previous studies have linked nursery supermarket access to the types of foods served.

The aim of this study was to examine the association between distance to the nearest supermarket and provision of fruits and vegetables in early years childcare, using data from a large, representative survey of English nurseries.

## 2 Materials and methods

### 2.1 Study sample

We used data from the Nutrition in Nurseries study, which surveyed by post a stratified random sample of 2000 nurseries across England in late 2012 and early 2013, with a response rate of 54% (851 nurseries). Nurseries were sampled from a list provided by Ofsted (the regulatory body for early year's childcare in England), which contained details of all organisations providing care for more than two hours per day and for more than six days per year. After-school and sports clubs, as well as childminders, were not eligible for inclusion in the study, nor were nurseries serving less than one snack or meal per day. Further details about the Nutrition in Nurseries study have been published previously (Neelon et al., 2015). All study procedures were approved by the University of Cambridge Psychology Research Ethics Committee.

### 2.2 Exposure – distance to nearest supermarket

We mapped nursery locations according to their reported postcode using a geographic information system (ArcGIS 10; ESRI., Redlands CA). Postcodes in the UK contain 15 addresses on average, and so allow for relatively precise geocoding (Smith et al., 2013, Burgoine et al., 2014). We sourced the locations of supermarkets belonging to major UK supermarket chains in the form of latitude and longitude coordinates with a stated spatial resolution of within 1 m, from Ordnance Survey (OS) Points of Interest (POI) data in July 2015. Containing data from over 170 suppliers (Ordnance Survey, 2008), OS POI data includes the locations of a wide range of public and private facilities across the UK. For the locations of supermarkets, OS POI data has been validated for use in food environments research against accurate food outlet data from English Local Authorities, demonstrating a 76% positive predictive value and 77% (‘good’) sensitivity (Burgoine and Harrison, 2013). These values are typical of those reported in validation studies of commercially available business data for food environments research, according to a recent meta-analysis (Lebel et al., 2017). We defined supermarkets as major chain retailers who held a substantial share of the UK grocery market at the time of the study: Tesco, Sainsbury's, ASDA, Morrisons, Waitrose, Aldi, and Co-operative (Kantar Worldpanel, 2016). Using OS MasterMap Integrated Transport Network data and the ArcGIS Network Analyst, we calculated distance from each nursery to the nearest supermarket (km) along the shortest street network route.

### 2.3 Outcome – frequency of serving fruits and vegetables

In the Nutrition in Nurseries study, managers were asked “how often [their] nursery serves these foods: a) fruits (not including 100% fruit juice); b) vegetables (not chips)”. The validity, test-retest reliability and inter-rater reliability associated with responses to these questions in a childcare setting has been previously established and reported (Benjamin et al., 2007). Response options were “never”, “less than once per week”, “once per week”, and “2–3 times per week or more”. For analysis here, we dichotomised response frequencies, with infrequent provision defined as serving *both* fruits and vegetables less than 2–3 times per week.

### 2.4 Covariates

The following variables were reported by nursery responders and considered as potential confounders: number of children enrolled in the nursery (continuous); number of years the nursery has been in operation (years, months); those with primary responsibility for preparing meals (nursery staff member vs non-nursery staff member (includes food service programs of a school or local school authority, a food service company or vendor, parents, others)); nursery manager's highest level of educational attainment (responses grouped as follows: compulsory education - none, GCSEs, NVQs; further education - A levels, 2-year diplomas; higher education - degrees, higher degrees); area-level deprivation (tertiles of index of multiple deprivation (IMD) 2010 scores for English lower super output areas (LSOAs)). Nursery responders indicated that in addition to supermarkets, they also purchased foods from convenience stores, specialist food retailers and wholesalers, proximity to which may serve as potential confounders. Therefore, we used OS POI food outlet location data to calculate street network distance (km) from each nursery to the nearest convenience store (POI use codes: 9470699, 10540737), specialist food retailer (9470662, 9470665, 9470666, 9470667, 9470668, 9470669, 9470670, 9470672, 9470705, 9470819) and wholesaler (9470768), for inclusion as additional covariates.

### 2.5 Statistical analysis

We used multiple binary logistic regression models to examine associations between distance to the nearest supermarket (quintiles) and odds of serving fruits and vegetables infrequently (both <2–3 times/week). Model 1 was our unadjusted model. Subsequent adjusted models included the covariates previously described in two groups: Model 2 included the number of children enrolled in the nursery, number of years the nursery had been in operation, those with primary responsibility for preparing meals, nursery manager's highest level of educational attainment, and area-level deprivation; Model 3 additionally adjusted for street network distances to other types of food outlet. This was a complete case analysis, with our sample limited to those with complete data across all variables of interest (n=623). As a sensitivity analysis, we performed multiple imputation utilising all of the Nutrition in Nurseries study data (n=850), with fully conditional specification, with 10 imputations, and which included the outcome and all covariates. As a second sensitivity analysis, we ran an alternative Model 2 specification without adjustment for area-level deprivation, including only the nursery manager's highest level of educational attainment by way of adjustment for SES. Results however were not substantively different from Model 2 as specified and are not presented. Data were analysed using PASW Statistics (SPSS Inc., Chicago IL) and SAS version 9.4 (SAS Institute, Cary NC), with a 2-tailed *P* ≤ 0.05 considered statistically significant.

## 3 Results

Descriptive statistics, overall and stratified by quintiles of distance to nearest supermarket are shown in Table 1. Nurseries in the study had been operating for a mean (SD) of 17 (12) years, had 55 (42) children enrolled, and were mostly managed by an individual with >13 years of education (58%). Nurseries with shorter distance to their nearest supermarket tended to be in more deprived areas. Mean (SD) distance to the nearest supermarket was 1.5 km (2.1 km), and nurseries with shorter distance to their nearest supermarket tended to be nearer to other types of food outlets.

**Table 1:** Characteristics of nurseries and nursery managers in the Nutrition in Nurseries sample (n=623), by street network distance to the nearest supermarket.

	**Distance to nearest supermarket quintile (Q)**^a^					**All**, n=623
	**Q1**, n=124	**Q2**, n=125	**Q3**, n=125	**Q4**, n=125	**Q5**, n=124	
	(0.0–0.4 km)	(0.4–0.6 km)	(0.6–1.0 km)	(1.0–1.7 km)	(1.7–19.8 km)	
Number of children enrolled per nursery (mean (SD))	56.0 (37.7)	57.7 (40.9)	59.7 (44.0)	55.5 (37.8)	46.3 (48.2)	55.1 (42.0)
Nursery years of operation (mean (SD))	17.3 (13.2)	17.0 (12.4)	16.7 (11.0)	14.8 (10.9)	18.8 (12.6)	16.9 (12.1)
Those primarily responsible for preparing meals (n (%)):^b^						
Nursery staff	84 (67.7)	86 (68.8)	85 (68.0)	83 (66.4)	74 (59.7)	412 (66.1)
Non-nursery staff	40 (32.3)	39 (31.2)	40 (32.0)	42 (33.6)	50 (40.3)	211 (33.9)
Nursery manager's highest educational attainment (n (%)):						
None/compulsory (≤11 years of education)	7 (5.7)	9 (7.2)	7 (5.6)	8 (6.4)	7 (5.7)	38 (6.1)
Further (12–13 years of education)	48 (38.7)	41 (32.8)	44 (35.2)	47 (37.6)	47 (37.9)	227 (36.4)
Higher (>13 years of education)	69 (52.7)	75 (60.0)	74 (59.2)	70 (56.0)	70 (56.5)	358 (57.5)
Nursery IMD deprivation tertile (n (%)):^c^						
Low (least deprived)	17 (13.7)	29 (23.2)	22 (17.6)	39 (31.2)	54 (43.6)	161 (25.8)
Middle	32 (25.8)	29 (23.2)	27 (21.6)	31 (24.8)	51 (41.1)	170 (27.3)
High (most deprived)	75 (60.5)	67 (53.6)	76 (60.8)	55 (44.0)	19 (15.3)	292 (46.9)
Street network distance (km) to nearest:						
Supermarket (mean (SD))	0.2 (0.1)	0.5 (0.1)	0.8 (0.1)	1.3 (0.2)	4.5 (3.1)	1.5 (2.1)
Specialist food store (mean (SD))	0.6 (0.7)	0.8 (0.7)	0.8 (0.7)	1.1 (0.7)	2.6 (1.8)	1.2 (1.2)
Convenience store (mean (SD))	0.3 (0.2)	0.4 (0.4)	0.4 (0.3)	0.6 (0.7)	1.6 (1.4)	0.7 (0.9)
Food wholesaler (mean (SD))	5.8 (6.8)	4.9 (4.7)	5.7 (6.0)	5.5 (5.3)	11.7 (7.9)	6.7 (6.7)
Nurseries serving fruits and vegetables less than 2–3 times per week (n (%))^d^	16 (12.9)	26 (20.8)	21 (16.8)	22 (17.6)	31 (25.0)	116 (18.6)

a Street network distance to the nearest supermarket (km), quintiles (Q): Q1=quintile with shortest distance to the nearest supermarket – Q5=quintile with longest distance to the nearest supermarket.

b Non-nursery staff include food service programs of a school or local school authority, a food service company or vendor, parents, others.

c Tertiles of index of multiple deprivation (IMD) 2010 scores for English lower super output areas (LSOAs). IMD is a compound measure of deprivation, capturing seven principle domains: income deprivation, employment, crime, health and disability, education, skills and training deprivation, barriers to housing and services and living environment deprivation.

d Nurseries were asked to indicate how often they served fruit (not including 100% fruit juice) and vegetables (not chips). Possible responses were ‘Never’, ‘Less than once per week’, ‘Once per week’ and ‘2–3 times per week or more’.

Overall, 116 (18%) nurseries served fruits and vegetables infrequently (both <2–3 times/week), however, this lack of provision differed by distance to the nearest supermarket. Nurseries with the farthest distance to their nearest supermarket were more likely to serve both fruits and vegetables infrequently (25%), relative to those with the least distance (13%).

In unadjusted and adjusted models (Table 2), distance from nursery to the nearest supermarket was significantly associated with fruit and vegetable serving frequency. In the unadjusted model 1, nurseries with farthest distance to their nearest supermarket (Q5) had 2.25 (95% CI 1.16–4.37) times the odds of serving fruits and vegetables infrequently compared to those nurseries with closest distance to their nearest supermarket (Q1). Adjusting for additional covariates including area-level deprivation (model 2) attenuated this association (Q5 vs Q1, OR=2.28 95% CI 1.11–4.68). In model 3, which also adjusts for proximity to other food outlets, those nurseries with farthest distance to their nearest supermarket (Q5) had 2.38 (95% CI 1.01–5.63) times the odds of serving both fruits and vegetable infrequently. In the maximally adjusted multiple imputation model, the estimates were attenuated but remained similar (Q5 vs Q1, OR = 2.24, 95% CI 0.86–5.85). Throughout, intermediate quintiles of supermarket proximity were not significantly associated with fruit and vegetable serving frequency.

**Table 2:** Associations of quintiles of distance to the nearest supermarket with odds of serving fruits and vegetables infrequently (both <2–3 times/week), estimated using a multiple binary logistic regression model in the Nutrition in Nurseries analytic sample (n =623).

		**Model 1**^a^		**Model 2**^b^		**Model 3**^c^	
	**Quintile (min-max)**	**OR**	**95% CI**	**OR**	**95% CI**	**OR**	**95% CI**
Distance to nearest supermarket quintile^d^	Q1 (0.0–0.4 km)	Ref		Ref		Ref	
	Q2 (0.4–0.6 km)	1.77	0.90–3.50	1.97	0.98–3.97	1.97	0.98–3.98
	Q3 (0.6–1.0 km)	1.36	0.67–2.76	1.50	0.73–3.08	1.50	0.73–3.10
	Q4 (1.0–1.7 km)	1.44	0.72–2.90	1.60	0.76–3.30	1.62	0.78–3.36
	Q5 (1.7–19.8 km)	2.25*	1.16–4.37	2.28*	1.11–4.68	2.38*	1.01–5.63

* *P*<0.05.

a Model 1 is an unadjusted model.

b Model 2 adjusts for number of children enrolled in the nursery, nursery years of operation, whether or not nursery staff were primarily responsible for preparing meals within nurseries, nursery manager's highest educational attainment and deprivation tertile of nursery lower super output area.

c Model 3 additionally adjusts for street network distance from nursery to nearest: specialist food store, convenience store, food wholesaler.

d Q1=quintile with shortest distance to the nearest supermarket – Q5=quintile with longest distance to the nearest supermarket.

## 4 Discussion

Using data from a national survey of English nurseries, we demonstrated a significant association between supermarket proximity and the provision of fruits and vegetables to children. Relative to nurseries located closest to their nearest supermarket, those farthest away had over twice the odds of serving fruits and vegetables infrequently. To our knowledge, this is the first study linking supermarket proximity, as a proxy for access to a range of affordable, healthy produce, to foods served in childcare settings.

There is a growing international body of literature suggesting that for adults, neighbourhood food retail, including supermarket access, is associated with diet, body weight and health (Cobb et al., 2015, Black et al., 2013, Caspi et al., 2012). In children, convenience store and fast food access have been associated with diet and obesity, but evidence for an association with supermarket access is more equivocal (Cobb et al., 2015, Fleischhacker et al., 2011). The majority of supermarket access studies in children, including those with fruit and vegetable consumption outcomes, have demonstrated a null association (Cobb et al., 2015). However, while these studies share methodological weaknesses, conceptually, these null associations might be better explained by young children's lack of autonomy to engage with supermarkets – specifically, whether to visit them or not. This may be less true of other food outlet types, for example fast food outlets, which hold broader appeal among children for economic and social reasons among others (Shift, 2013, Timperio et al., 2008, Shephard et al., 2006). Although untested, we suggest that the importance of supermarket access to young children is not by way of their own proximity, but is rather mediated through use of supermarkets by those (adults) with a responsibility of care; parents, guardians, or here, those responsible for food provision in nurseries. This hypothesis suggests that the fairest comparison for our results is with studies examining the implications of supermarket access for diet in adults; a literature base that is well developed, but has not yet focused on the behaviours of those in childcare.

Unlike in the US and Canada, there are few formal recommendations to guide nursery food provision in the UK, with statutory guidance mandating only that meals, drinks and snacks served in nurseries are ‘healthy, balanced and nutritious’ (EYFS Review, 2011). Our results are noteworthy because they suggest that supermarket access may be important for nurseries in this regard. However, our results do not speak to the ability of supermarkets to help nurseries meet specific voluntary guidelines for the provision of fruits and vegetables to children, such as those established by the Children's Food Trust. These guidelines, which were supported by Public Health England in the 2016 UK Childhood Obesity Strategy (HM Government, 2016), recommend nurseries serve five varied portions of fruits and vegetables per day, with at least one portion served at each meal and with some snacks (Children's Food Trust, 2010b). The role of supermarkets in supporting these specific recommendations should be the focus of future research.

### 4.1 Limitations

Our study has a number of limitations, the most notable being the observational, cross-sectional study design, which restricts causal inference. The relationships observed may reflect the desire of nurseries who intend to serve fruits and vegetables frequently to locate in close proximity to a supermarket, or, the opening of a supermarket in close proximity to a nursery that is already serving fruits and vegetables frequently. Exposure misclassification may have resulted from what has been termed the ‘uncertain geographic context problem’ (Kwan, 2012). We assumed that supermarkets would be accessed from the nursery, however these visits could have been made from other locations, such as from the homes of nursery staff. We also assumed that grocery shopping would be undertaken in-store, and not via delivery services such as those offered by major English supermarket chains, which may minimise the importance of supermarket proximity. Further, our analysis did not account for economic factors. Notably, the provision of healthier foods in childcare settings is associated with higher food expenditures (Monsivais and Johnson, 2011), and the higher prices at some supermarket chains may make these outlets less accessible to some consumers, even if they are geographically close by (Mackenbach et al., 2017). However, our inability to account for these factors is not a limitation unique to our study (Charreire et al., 2010). Moreover, utilisation of ‘online’ grocery delivery services in England remains limited, with sales accounting for only 6% of the total grocery sector (Mintel, 2016). Exposure misclassification elsewhere was minimized (although not eliminated) through use of accurate secondary food outlet data (Burgoine and Harrison, 2013, Lebel et al., 2017), and through minimization of temporal mismatch, as exposures (supermarket locations in 2015) were measured soon after outcomes (fruit and vegetable consumption frequency in 2012/13).

Nursery managers reported how often fruits and vegetables were served, however, reported estimates may be subject to biases, including social desirability bias; and fruit and vegetable *provision* may not reflect fruit and vegetable *consumption*. This said, the extent to which such errors were systematic across supermarket distance quintiles, and therefore impacting our findings is unclear. Moreover, within schools and nurseries, greater availability of fruits and vegetables has been linked consistently to greater fruit and vegetable consumption (French and Stables, 2003; Ball et al., 2008). Over 80% of nurseries in our analytic sample served fruit and vegetables 2–3 times per week or more; we were unable to test higher serving frequency thresholds due to the instrument used in the Nutrition in Nurseries survey to capture fruit and vegetable provision. Due to further limitations of the survey, we were only able to assess provision of fruits and vegetables, and were unable to evaluate the overall quality of foods served. Lastly, although we acknowledge the 54% response rate achieved in the Nutrition in Nurseries study, which could limit generalisability, the nurseries were distributed relatively evenly across the country and socioeconomic strata therein and this response rate is consistent with other surveys of early years childcare settings (United States Department of Agriculture: Food and nutrition service, 2013, Organix and Soil Association, 2008). Furthermore, the nurseries included in this study were representative of all English nurseries listed by Ofsted in terms of mean distance (km) from each to their nearest supermarket (analytic sample (n = 623), mean(SD) = 1.46 (2.10); all nurseries (n = 28,091), 1.58 (2.09)). This reduces the potential for bias in the relationship observed between those nurseries who did and did not participate in the Nutrition in Nurseries study.

The major strength of this study is the use of a national survey of early years child care providers, with good representation from across the socioeconomic spectrum. The is the largest survey of its kind in England, which combined with national food outlet location data, allowed for the first assessment of the relationship between supermarket access and fruit and vegetable provision in nurseries.

## 5 Conclusions

In England, nurseries are widely-attended by children from across the socioeconomic spectrum, and their importance in promoting consumption of healthy foods during childhood and across the lifecourse is well recognised (Marmot, 2010). Therefore, understanding the barriers and enablers to healthy food provision in these settings is critical. This study used data from a large, nationwide survey of nutrition practices in English nurseries, in combination with accurate food outlet location data. For the first time in the published literature, we established a relationship between physical supermarket access and fruit and vegetable provision in nurseries. Our study is important because it adds to a wide and growing body of literature, which has demonstrated this association for individuals but not as yet for the practices of institutions. Our results suggest that supermarket access may be important for nurseries in meeting guidelines for the provision of fruits and vegetables to help promote healthy eating in young children.

## Conflicts of interest

None.

## Ethical approval

All study procedures were approved by the University of Cambridge Psychology Research Ethics Committee.
